# Supplementary material for: Content-rich biological network constructed by mining PubMed abstracts
Source: BMC Bioinformatics. 2004 Oct 8;5:147. doi: 10.1186/1471-2105-5-147 (PMC528731; doi:10.1186/1471-2105-5-147)
Supplement: Additional File 2 — The original results of the above study (non-essential files are deleted to keep the file size under the limit set by BMC bioinformatics). [file 1471-2105-5-147-S2.bz2 › chilibotAdditionalFile2/dip50/402ID10026196E10012/html/IL11_IL6ST.html]

 


 **IL11** and **IL6ST** 
  
Found 219 abstracts in PubMed, retrieved 50.  
 

 What does Google say? 
 PDF only 
| .edu only 

---

**Interactive relationship** (e.g. stimulation, inhibition, etc)

**Inhibitory relationship**- Here we show that MDL A has a highly selective inhibitory effect on IL 6 and IL 11  [ **IL11** ]  activities by inhibiting a gp130  [ **IL6ST** ]  activity while suppressing bone loss in ovariectomized mice.  Ref: 12417753 Proc Natl Acad Sci U S A, 2002
- Osteoclastogenesis induced by IL 6 and IL 11  [ **IL11** ]  was inhibited by the addition of an anti gp130  [ **IL6ST** ]  antibody but not by osteoprotegerin.  Ref: 12584029 Bone, 2003
- Resorption caused by IL 11  [ **IL11** ]  was also inhibited by both anti mouse glycoprotein 130  [ **IL6ST** ]  and an antibody neutralizing IL 11  [ **IL11** ] , but these agents had no effect on Ca release caused by PTH or 5vitamin D.  Ref: 12110441 Bone, 2002
- gp130  [ **IL6ST** ]  either homodimerizes in response to IL 6 and IL 11  [ **IL11** ]  or forms heterodimers with the leukemia inhibitory factor LIF receptor LIFR in response to LIF, oncostatin M OSM, ciliary neurotrophic factor CNTF, cardiotrophin 1 CT 1 or cardiotrophin like cytokine resulting in the onset of cytoplasmic tyrosine phosphorylation cascades.  Ref: 12047380 Eur J Biochem, 2002
- Here we show that Cdk9 interacts with gp130  [ **IL6ST** ] , the receptor of the Interleukin 6 IL 6 family of cytokines, which includes Leukemia Inhibitory Factor LIF, Oncostatin M OSM, Ciliary Neurotrophic Factor CNTF, Interleukin 11  [ **IL11** ]  IL 11  [ **IL11** ]  and Cardiotrophin CT 1.  Ref: 12386808 Oncogene, 2002
**Neutral relationship**- IL 11  [ **IL11** ]  belongs to the gp130  [ **IL6ST** ]  cytokine family, which includes also IL 6.  Ref: 12697042 J Endocrinol, 2003
- IL 11  [ **IL11** ]  forms a high affinity complex, thereby inducing gp130  [ **IL6ST** ]  dependent signaling.  Ref: 12865319 Endocrinology, 2003
- IL 11  [ **IL11** ]  acts via a heterodimeric receptor composed of a ligand specific receptor alpha chain IL 11R alpha and the signalling moiety gp130  [ **IL6ST** ] .  Ref: 11570967 Reproduction, 2001
- IL 11  [ **IL11** ]  is a member of the gp130  [ **IL6ST** ]  family of cytokines, which signal via assembly of multisubunit receptor complexes containing at least one molecule of the transmembrane signaling receptor gp130  [ **IL6ST** ] .  Ref: 12865319 Endocrinology, 2003
- The levels of gp130  [ **IL6ST** ]  were significantly stimulated by the addition of interleukin 1  [ **IL11** ]  beta IL 1  [ **IL11** ]  beta or interleukin 6 IL 6 but not by the addition of follicle stimulating hormone FSH.  Ref: 12108784 Endocr Res< MedlineTA>Endocr Res, 2003

**Non-interactive relationship** (e.g. studied together, co-existance, homology, etc.)

- Interleukin IL 6, leukaemia inhibitory factor LIF and IL 11  [ **IL11** ]  belong to the same family of cytokines whose receptors utilize gp130  [ **IL6ST** ]  as the signalling molecule.  Ref: 12200462 Mol Hum Reprod, 2002
- This study examined the expression of IL 11  [ **IL11** ] , IL 11Ralpha, and signal transduction factor glycoprotein 130  [ **IL6ST** ]  in different phases of endometrium six in proliferative phase and four in secretory phase, and the decidua and villi of normal pregnancy NP.  Ref: 11994383 J Clin Endocrinol Metab, 2002
- The functional receptor complex comprises IL 11  [ **IL11** ] , IL 11R and the signal transducing subunit gp130  [ **IL6ST** ] .  Ref: 11237599 J Mol Biol, 2001
- Exposure of these cells to other gp130  [ **IL6ST** ]  receptor family cytokines, including leukemia inhibitory factor LIF, interleukin 6 IL 6, and IL 11  [ **IL11** ] , do not significantly affect DNA synthesis or induction of tPA PAI 1.  Ref: 12090757 Cytokine, 2002
- Administration of either interleukin 6 or interleukin 11  [ **IL11** ]  to gp130  [ **IL6ST** ]  delta delta mice failed to increase platelet numbers, despite an increase in the production of megakaryocytes.  Ref: 12423677 Exp Hematol, 2002
- Leukemia inhibitory factor LIF, oncostatin M OSM, interleukin IL 6, and IL 11  [ **IL11** ]  regulate hemopoiesis and LIF, OSM, and IL 6 also inhibit the proliferation of myeloid leukemic cell lines through the signal transducing subunit gp130  [ **IL6ST** ] .  Ref: 12543079 Cytokine, 2002
- Effects of the gp130  [ **IL6ST** ]  cytokines ciliary neurotropic factor CNTF and interleukin 11  [ **IL11** ]  on pituitary cells CNTF receptors on human pituitary adenomas and stimulation of prolactin and GH secretion in normal rat anterior pituitary aggregate cultures.  Ref: 11375124 J Endocrinol, 2001
- ELISA was used to determine the serum levels of soluble interleukin 2 receptors, interleukin 1  [ **IL11** ] , interleukin 6, and 2 soluble interleukin 6 receptors sIL 6R and soluble gp130  [ **IL6ST** ]  in 18 patients with cluster headache 6 women and 12 men during the cluster period and in 17 healthy controls who were headache free 3 women and 14 men.  Ref: 12864761 Headache, 2003
- These findings indicate that IL 2 stimulated NK cells produce a novel peptide, distinct from TPO, IL 6, IL 11  [ **IL11** ] , LIF, other gp130  [ **IL6ST** ]  associated interleukins, and SDF1a, that can induce in vitro endomitosis in immature human megakaryocytes in the presence of IL 3 and SCF.  Ref: 11756162 Blood, 2002
- In liquid cultures, megakaryocytes from gp130  [ **IL6ST** ]  delta delta mice are smaller than those from wild type mice and do not increase in size upon stimulation with interleukin 6 or interleukin 11  [ **IL11** ] .  Ref: 12423677 Exp Hematol, 2002
- These and others like IL 11  [ **IL11** ]  and ciliary neurotrophic factor are referred to as the gp130  [ **IL6ST** ]  cytokines because they share the gp130  [ **IL6ST** ]  glycoprotein as a common receptor initial signal transducer.  Ref: 12538632 Endocrinology, 2003
- Recent investigations of the cytokine network surrounding myeloma cells have disclosed the importance of gp130  [ **IL6ST** ]  related cytokines including interleukin IL 6 for myeloma cell survival and proliferation, identification of IL 10 as a growth factor for myeloma cells, the close relationship between IL 10 and the receptors for gp130  [ **IL6ST** ]  related cytokines, and the growth enhancement effect of IL 11  [ **IL11** ]  and IL 7 on myeloma cells.  Ref: 11122145 Br J Haematol, 2000
- We observed that, in addition to up regulation of IL 1beta and to a lesser extent TNF alpha, IRI was associated with an intense and sustained up regulation of three gp130  [ **IL6ST** ]  signaling cytokines, IL 6, IL 11  [ **IL11** ] , and leukemia inhibitory factor LIF, as well as with up regulation of the neutrophil chemotactic and activating mediator macrophage inflammatory protein MIP 2.  Ref: 10755557 Transplantation, 2000
- Cytokines belonging to the so called interleukin 6 IL 6 or gp130  [ **IL6ST** ]  cytokine family, notably IL 6 and IL 11  [ **IL11** ] , are known as pro resorptive cytokines, in that they promote osteoclastogenesis.  Ref: 12114264 Ann N Y Acad Sci, 2002
- In 45 primary ovarian carcinoma samples where both components of the IL 11  [ **IL11** ]  receptor IL 11Ralpha and gp130  [ **IL6ST** ]  were examined, coexpression was observed in 42 93.3%.  Ref: 11161848 Gynecol Oncol, 2001
- Semiquantitative reverse transcription polymerase chain reaction RT PCR analysis showed that IL 11  [ **IL11** ]  and its receptor, CNTF and its receptor, LIFR, and gp130  [ **IL6ST** ]  were constitutively expressed throughout the culture period.  Ref: 12110437 Bone, 2002
- In contrast, only mRNA for the IL 11  [ **IL11** ]  receptor and gp130  [ **IL6ST** ]  were detected in BMMC.  Ref: 11820727 Arch Dermatol Res, 2001
- The activity in NKCM was dose dependent and could not be neutralized by an excess of antibodies to IL 6, IL 11  [ **IL11** ] , leukemia inhibitory factor LIF, gp130  [ **IL6ST** ] , stromal cell derived factor 1a SDF 1a, and thrombopoietin TPO.  Ref: 11756162 Blood, 2002
- RESULTS Eighteen genes of 76 were found to be up regulated after 24 hours of infection with this obligate intracellular bacterium, among them the glycoprotein 130  [ **IL6ST** ]  family members IL 11  [ **IL11** ]  and LIF, the chemokine gene MIP2 alpha, the transcription factor genes EGR1, ETR101, FRA1, and c jun, the apoptosis related genes IEX 1L and MCL 1, adhesion molecule genes such as ICAM1, and various other functionally important genes.  Ref: 11665982 Arthritis Rheum, 2001
- Synergistic effects of glycoprotein 130  [ **IL6ST** ]  binding cytokines in combination with interleukin 1  [ **IL11** ]  on cartilage collagen breakdown.  Ref: 11465713 Arthritis Rheum, 2001
- OnM induced desensitization also prevents responses to other gp130  [ **IL6ST** ]  signaling cytokines e.g. leukemia inhibitory factor and interleukin 11  [ **IL11** ] .  Ref: 12724316 J Biol Chem< MedlineTA>J Biol Chem, 2001
- We have used measurements of extracellular acidification rate ECAR of HepG2 cells cultured on microporous membranes to probe the coupling between signaling pathways for gp130  [ **IL6ST** ]  family cytokines interleukin 6, oncostatin M and IL 1  [ **IL11** ] , each of which is considered to play a significant role in the hepatic acute phase response.  Ref: 11246741 Biophys Chem, 2001
- We demonstrate here that expression of gp130  [ **IL6ST** ]  and IL 11  [ **IL11** ]  receptor IL 11R alpha mRNA, components of the IL 11R complex, are detected in human and murine CD4 and CD8 lymphocytes, suggesting that rHuIL 11 can directly interact with T cells.  Ref: 11177577 J Interferon Cytokine Res, 2001
- Cytokines including IL 1  [ **IL11** ] , TNF, and members of the gp130  [ **IL6ST** ]  cytokine family participate as mediators of a complex HPA axis response to stress and inflammation.  Ref: 11956136 Endocrinology, 2002
- OBJECTIVE The development of megakaryocytes is regulated by thrombopoietin TPO, which binds to the c mpl receptor, and by several other cytokines such as interleukin IL 6, IL 11  [ **IL11** ] , leukemia inhibitory factor LIF, cilliary neurotropic factor CNTF, and oncostatin OSM, which bind to gp130  [ **IL6ST** ]  protein coupled receptors.  Ref: 12135673 Exp Hematol, 2002
- Also, the effect of BMP 6 on marrow stroma production of interleukin 1  [ **IL11** ]  and their common receptor gp130  [ **IL6ST** ]  that is expressed in haemopoietic stem cells and is indispensable for their proliferation and tri lineage differentiation was examined.  Ref: 11401330 Cell Biol Int, 2001
- RT PCR showed IL 11  [ **IL11** ] , IL 11Ralpha, and glycoprotein 130  [ **IL6ST** ]  mRNA expression in all samples, except the absence of IL 11  [ **IL11** ]  signal in the unstimulated MRC 5 cell and the proliferative phase endometrium.  Ref: 11994383 J Clin Endocrinol Metab, 2002
- The binding of 32 P labeled FP Delta IL 11  [ **IL11** ]  to Ba F3 cells stably transfected with plasmids encoding human IL 11  [ **IL11** ]  receptors alpha and beta chains IL 11R alpha and gp130  [ **IL6ST** ]  was specific and saturable with a high affinity as determined from Scatchard plot analysis.  Ref: 11784299 Eur J Biochem, 2002
- Recombinant human Interleukin 11  [ **IL11** ]  rhIL 11, Neumega TM, a gp130  [ **IL6ST** ]  signalling cytokine approved for treatment of thrombocytopenia, also raises von Willebrand factor VWF and factor VIII FVIII by an unknown mechanism.  Ref: 12649145 Blood< MedlineTA>Blood, 2002
- OBJECTIVES The objectives of this study were to determine 1 the frequency of expression of the interleukin 11  [ **IL11** ]  receptor alpha subunit IL 11Ralpha and its signal transducing subunit, gp130  [ **IL6ST** ] , among primary ovarian carcinomas.  Ref: 11161848 Gynecol Oncol, 2001
- Continuous treatment with Dex 10 8 mol L inhibited the endogenous production of IL 6, LIF, OSM, IL 11R, and OSMR, but had no detectable effect on the expression of IL 11  [ **IL11** ] , CT 1, CNTF, CNTFR, LIFR, or gp130  [ **IL6ST** ] .  Ref: 12110437 Bone, 2002
- To our surprise, none of the other gp130  [ **IL6ST** ]  protein related cytokines tested IL 11  [ **IL11** ] , LIF, CNTF, and OSM activated these signaling pathways in CD34 cells, megakaryoblasts, or platelets.  Ref: 12135673 Exp Hematol, 2002
- Previous studies have identified three distinct receptor binding sites, I, II, and III, crucial for the binding of murine IL 11  [ **IL11** ]  mIL 11 to both the IL 11R and gp130  [ **IL6ST** ] .  Ref: 12865319 Endocrinology, 2003
- We have therefore evaluated, by immunohistochemical image analyses, time course changes of interleukin IL 1beta, IL 6, tumor necrosis factor TNF alpha, IL 6 receptor gp130  [ **IL6ST** ] , IL 1  [ **IL11** ]  receptor I, and cyclooxygenase Cox 2 protein in brain cortex, hippocampus and the ventromedial hypothalamic nucleus VMH in tumor bearing mice with prostanoid related anorexia.  Ref: 11406541 Cancer Res, 2001
